# Ultrasonographic and elastographic findings of the liver of dogs exposed to cigarette smoke

**DOI:** 10.29374/2527-2179.bjvm005026

**Published:** 2026-07-24

**Authors:** Felipe Gasparini Echem Gomes, Camila Silveira Stanquini, Giovanna Serpa Maciel Feliciano, Vitoria de Oliveira Rodrigues, Gabriel Sousa Santos, Luiz Paulo Nogueira Aires, Anna Carolina Mazeto Ercolin, Sâmara Turbay Pires, Javier Enrique Jerez Arevalo, Fernando Sebastián Baldi Rey, Carlos Eduardo Ambrósio, Marcus Antônio Rossi Feliciano

**Affiliations:** 1 Programa de Aprimoramento Profissional em Medicina Veterinária - Diagnóstico por Imagem, Departamento de Medicina Veterinária (ZMV), Faculdade de Zootecnia e Engenharia de Alimentos (FZEA), Universidade de São Paulo (USP), Pirassununga, SP, Brazil.; 2 Programa de Pós-Graduação em Biociência Animal (PPGBIO), ZMV, FZEA, USP, Pirassununga, SP, Brazil.; 3 Programa de Pós-Graduação em Ciências Veterinárias (Saúde Animal), Faculdade de Ciências Agrárias e Veterinárias (FCAV), Universidade Estadual Paulista “Júlio de Mesquita Filho” (UNESP), Jaboticabal, SP, Brazil.; 4 Independent veterinarian, Pirassununga, SP, Brazil.; 5 ZMV, FZEA, USP, Pirassununga, SP, Brazil.

**Keywords:** B-mode ultrasound, dog, SWE elastography, tobacco, ultrassom em modo-B, cão, elastografia SWE, tabaco

## Abstract

Smoking is identified as one of the main causes of non-transmissible diseases, damaging organs such as the lungs, liver, kidneys, and heart. Owing to their proximity to smokers, dogs are passively exposed to substances present in cigarette smoke. We aimed to describe the ultrasonographic and elastographic findings of the livers of dogs exposed to cigarette smoke. The study involved 13 dogs chronically exposed to cigarette smoke and 10 with no history of exposure to cigarette smoke or progressive liver changes in the preceding 6 months. The animals were subjected to laboratory testing, B-mode ultrasound, and Shear Wave elastography. Statistical analyses used a binomial model for categorical variables and a negative binomial or Poisson model for numerical variables, considering a p-value <0.05. Dogs exposed to tobacco smoke showed reduced liver stiffness. This result may be associated with a congestive process or fatty liver disease. Hepatic congestion may be associated with pulmonary hypertension caused by the inhalation of cigarette smoke. Exposure to tobacco smoke leads to the progression of hepatic fatty infiltration. However, histopathology and a detailed cardiopulmonary investigation are required to confirm these hypotheses. Dogs exposed to cigarette smoke tended to exhibit hepatomegaly and altered liver echotexture. Dogs over 5 years of age tended to show altered liver echogenicity. The B-mode ultrasonography and elastography findings suggest that passive exposure to cigarette smoke can adversely impact animal health. The findings may provide a basis for future investigations among relatively large and homogeneous populations.

## Resumo

O tabagismo é uma das principais causas de doenças não transmissíveis, lesionando órgãos como pulmão, fígado, rins e coração. A proximidade com humanos faz com que cães sejam expostos passivamente à fumaça de cigarro. O objetivo deste estudo é descrever os achados ultrassonográficos modo-B e elastografia do fígado de cães expostos à fumaça do cigarro. Este estudo envolveu 13 cães expostos cronicamente à fumaça de cigarro e 10 cães sem histórico de exposição à fumaça de cigarro ou de alterações hepáticas progressivas nos 6 meses precedentes. Os animais foram submetidos a exames laboratoriais, ultrassom modo-B e elastografia Shear Wave. O modelo binomial foi usado para variáveis categóricas e o modelo binomial negativo ou de Poisson para variáveis numéricas, considerando p-valor <0,05. Cães expostos apresentaram menor rigidez hepática, sugerindo possível associação à um processo congestivo ou infiltração gordurosa. A congestão hepática pode ser associada à hipertensão pulmonar pela inalação de fumaça de cigarro. A exposição à fumaça de cigarro também pode causar progressão da infiltração gordurosa hepática. Contudo, a análise histopatológica e uma investigação cardiopulmonar detalhada são necessárias para confirmação dessas hipóteses. Cães expostos tenderam a desenvolver hepatomegalia e alteração de ecotextura hepática. Cães com mais de cinco anos exibiram maior probabilidade para alterações de ecogenicidade hepática. Assim, os achados ultrassonográficos e elastográficos indicam que a exposição passiva ao cigarro pode comprometer a saúde dos animais. Os resultados apresentados podem futuramente embasar investigações em populações maiores e mais homogêneas.

## Introduction

Cigarettes are one of the most widespread legal drugs; their harmful effects on active and passive smokers have been studied extensively (Ka et al., 2014). Approximately 5,000 substances released in cigarette smoke resulting from the burning of tobacco leaves have been described. These substances form a complex and dynamic aggregate with hepatotoxic and carcinogenic properties, exerting systemic effects on various organs (lungs, kidneys, spleen, and liver) (Liang et al., 2022; Rosemberg, 2004; Talhout et al., 2011).

Exposure to smoke components occurs through inhalation via the lungs, metabolization in the liver, and excretion by the kidneys. Therefore, these organs are the primary targets of damage (Bandiera et al., 2020; Marti-Aguado et al., 2022). Although the liver does not come into direct contact with cigarette smoke, the effects of smoking or indirect exposure to cigarette smoke are mediated by the oxidative effect that its metabolites exert on liver parenchymal cells. This oxidative effect can trigger scarring/fibrosis responses. The impacts of smoking or exposure to cigarette smoke are also associated with the indirect oxidative effects resulting from the accumulation of pro-inflammatory cytokines and catabolic iron levels. These indirect effects exacerbate the primary oxidative response (El-Zayadi, 2006).

Similar to humans, pets living with smokers are passively exposed to cigarette smoke via inhalation or, in certain cases, by ingesting residual particulate matter that has settled on surfaces and mixed with ambient dust. This exposure is increased in dogs that habitually rest on surfaces such as carpets and rugs, which results in increased contact with particulate matter from cigarette smoke. Additionally, behavioral traits such as sniffing and anatomical and physiological characteristics such as the superior proximity of the upper airways to the ground and a relatively high respiratory rate make dogs increasingly susceptible to inhaling residual particles present in the environment shared with a smoker (Roberts & Dickey, 1995).

Liver lesions such as fibrosis, neoplasms, or areas of necrosis can be assessed using histological and biochemical methods (Bandiera et al., 2020; Lawrence & Steiner, 2017). However, imaging studies can serve as important diagnostic tools for evaluating liver function in these patients. Conventional ultrasound (B-mode) is commonly used in the evaluation of liver diseases (Jeon et al., 2015). This imaging modality is notable for its high sensitivity in detecting focal and diffuse abnormalities in the liver parenchyma, ease of application, non-invasive nature, and absence of ionizing radiation exposure (Al Salmi et al., 2021; Leela-Arporn et al., 2021). Furthermore, this technique can aid in the early diagnosis of liver diseases, considering that the clinical manifestations of liver injury in early stages can be nonspecific and most clinical signs become evident only after a significant degree of liver impairment. However, B-mode ultrasound alone is limited in these cases; its specificity remains controversial across studies, which may compromise its diagnostic effectiveness (Jeon et al., 2015; Manica & Martins, 2010).

To mitigate the limitations of B-mode ultrasonography, it can be complemented by advanced imaging modalities, such as elastography. Elastography is an important diagnostic tool for identifying possible changes in soft tissue elasticity, such as aging/fibrosis, inflammation, and malignancy in neoplasms. This technique has been largely used for the assessment of lesions in the liver (Emara et al., 2019; Feng et al., 2016; Jeon et al., 2015), breast (Feliciano et al., 2014; Zhou et al., 2013), thyroid (Ramos et al., 2024), prostate (Cintra et al., 2020; Jeon et al., 2015), adrenal (Alves et al., 2024), lung (Lima et al., 2025), kidney (Moraes et al., 2025), and lymph nodes (Favril et al., 2022), with proven efficacy in numerous studies in human and veterinary medicine (Gennisson et al., 2013; Sigrist et al., 2017).

Shear wave elastography (SWE) has a sensitivity of 0.78 and specificity of 0.84 for the detection of liver fibrosis in humans (Bota et al., 2013). In dogs, SWE has also demonstrated an increase in shear velocity while evaluating the fibrotic tissue (Facin et al., 2020). This result indicates increased stiffness in the analyzed tissue, possibly associated with an elevated incidence of scarring in the evaluated organ (Yoneda et al., 2010). Furthermore, elastography is an excellent technique for differentiating malignancy in neoplasms. Malignant lesions often exhibit greater stiffness than benign lesions (Jeon et al., 2015).

Regular cigarette smoking is directly associated with the extent of liver fibrosis in humans, as quantified by elastography based on tissue stiffness values (Ou et al., 2019). Tissue stiffness is measured quantitatively using shear wave velocity. In addition, SWE elastography can provide qualitative information through the elastogram, a static color-coded map indicating the relative stiffness of tissues, by comparing it with the conventional ultrasound image of the same evaluated area (Goddi et al., 2012; Ou et al., 2019; Sigrist et al., 2017).

The kidneys of dogs exposed to cigarette smoke were recently studied using B-mode ultrasound and elastography (Moraes et al., 2025). Ultrasonography demonstrated a significant increase in renal echogenicity and irregular contours in the exposed group; however, the exposed and unexposed groups showed no differences in echotexture, corticomedullary ratio, and the presence of abnormalities. In the elastographic assessment, tissues were relatively stiff in exposed dogs (p = 0.0492), indicating that exposure to secondhand smoke could adversely impact canine renal health, with early changes detectable by conventional ultrasound and elastography. Therefore, based on the above information, we aimed to describe the quantitative ultrasonographic and elastographic characteristics of liver tissue in dogs exposed to toxic substances present in cigarette smoke. This approach is intended to contribute to the early diagnosis of possible changes resulting from this exposure, thereby allowing for suitable clinical management and a potentially favorable prognosis for these patients.

## Materials and methods

This study was conducted at the Veterinary Hospital (HOVET) of the Faculty of Animal Science and Food Engineering of the University of São Paulo (FZEA/USP) and was approved by the Animal Ethics (CEUA-FZEA/USP) under protocol no. 5248010623. The animals included in this study were obtained from HOVET’s routine clinical practice or recruited from residents of the city of Pirassununga, São Paulo, Brazil and the surrounding region.

After physical examination and medical history review, 23 animals were divided into two groups: the exposed group, consisting of 13 dogs previously exposed to cigarette smoke for 2 years or longer and with no history or clinical signs consistent with liver disease, and the control group, consisting of 10 dogs with no history of cigarette smoke exposure and no liver abnormalities in the preceding 6 months. Dogs presenting jaundice or a recent history of clinical signs suggestive of acute hepatitis, such as vomiting, diarrhea, or weight loss, were excluded from the sample. After defining the groups, all animals were subjected to B-mode and elastographic ultrasound examinations in addition to blood collection for analysis of the hepatic biochemical markers alanine aminotransferase, total bilirubin, and alkaline phosphatase.

Prior to the imaging exams, the dogs were fasted for 8 h and had their abdominal hair clipped. The examinations were performed using an ESAOTE MX8® ultrasound system (Italy), employing microconvex (2.5-5 MHz) and linear (5-10 MHz) multi-frequency matrix transducers.

### B-mode ultrasound examination

The animals were positioned in the dorsal or lateral recumbent position and initially subjected to a B-mode ultrasound examination. The hepatic evaluation included echotexture (homogeneous or heterogeneous), echogenicity of the hepatic parenchyma compared with that of the right kidney and spleen (hyperechoic, isoechoic, or hypoechoic), size (normal, enlarged, or reduced), and contours (regular or irregular).

### Elastographic analysis

Hepatic elastography was performed to measure the velocity of acoustic propagation in the liver. Quantitative analysis consisted of measuring shear wave velocity, with at least five measurements taken per animal, expressed in meters per second (m/s); these values were compared between the control and exposed groups. Five samples (region of interest; ROI) were randomly collected from different portions and depths of the liver and used to calculate the means. The sample depth values were provided by the ultrasound device and subsequently used to assess the influence of this parameter on the results obtained. Discrepant values, attributed to artifacts or interference during image acquisition, were discarded to ensure superior accuracy of the results. These data were compared between the two sample groups to verify differences in image patterns related to smoke exposure.

### Analysis of smoke exposure

Additionally, the effect of the degree of smoke exposure on B-mode ultrasound and elastographic parameters was investigated. For this purpose, smoke exposure was categorized according to “Cigarettes Smoked Per Day” into unexposed (n = 10), low exposure (fewer than 10 cigarettes per day, n = 10), and high exposure (more than 10 cigarettes per day, n = 3). The “Exposure Duration” variable was divided into two groups using the mean exposure duration (5.43 years) as the cutoff point. The animals were categorized as unexposed (n = 10), high exposure (more than 5.43 years, n = 6), or low exposure (5.43 years or less, n = 7). The variable “Number of smokers” was divided into unexposed (n = 10), low exposure (only one person, n = 6), and high exposure (more than one person, n = 7). These values were compared with the variables of imaging findings and biochemical tests to identify possible correlations between cigarette smoke exposure variables and the identified liver changes.

Additionally, the effect of the age of the animals on the identified liver changes was analyzed. The animals were divided into two groups (up to 5 years, n = 8 and over 5 years, n = 15).

### Statistical analysis

The completely randomized design was analyzed using the PROC GENMOD procedure in SAS (SAS Institute, 2023), with a significance level of 5% for the tests.

For categorical variables, a generalized linear model was used, assuming a normal binomial distribution, with the control and exposed groups included as fixed effects.

A binomial model was used for the analysis of the categorical variables of age and those associated with the degree of exposure (number of smokers, number of cigarettes, and duration of exposure), considering age, number of smokers, number of cigarettes, and duration of exposure as independent variables.

The numerical data (stiffness and depth) with an asymmetric distribution were analyzed using the Poisson and Negative Binomial models.

## Results

**Figure 1:**
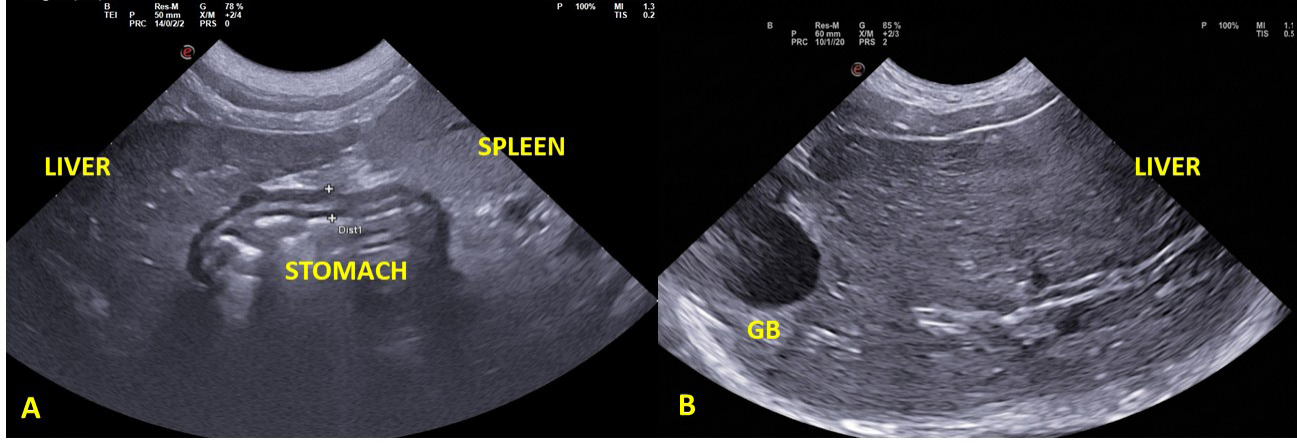
B-mode ultrasound of the liver in dogs. (A) Dog from the control group, not exposed to cigarette smoke, showing homogeneous liver parenchyma, less echoic than the spleen, and tapered edges; (B) Dog exposed to cigarette smoke. Note the inhomogeneous and echogenic appearance of the hepatic parenchyma and attenuation of the hepatic vessels. GB = Gallbladder.

**Figure 2:**
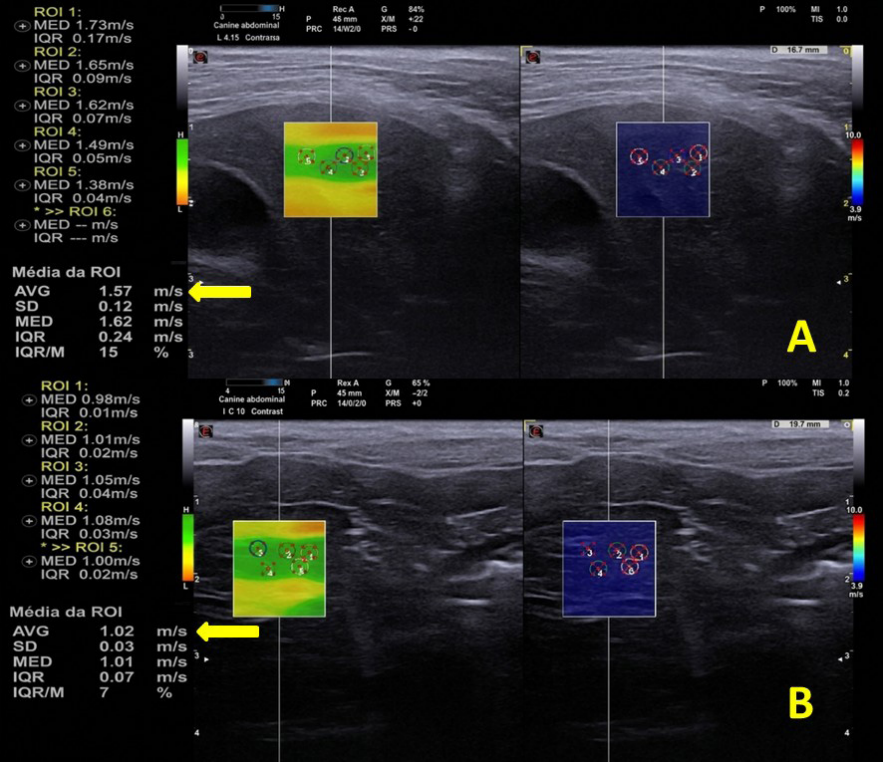
Qualitative and quantitative elastography of the liver in the control group (A) and exposed group (B). Note the average of the ROIs (AVG-arrow) indicating a shear velocity of 1.57 m/s in the control group and 1.02 m/s in the exposed group, along with bluish coloring on the elastogram, indicating relatively soft liver tissue.

Qualitatively, during B-mode ultrasound examination, differences between dogs in the control group and those in the exposed group could clearly be identified. Compared with those in the control group, dogs in the exposed group showed an enlarged liver with rounded edges, increased echogenicity of the hepatic parenchyma, coarse echotexture, and greater difficulty in identifying hepatic portal vessels (Figure 1). In the qualitative elastographic evaluation, a predominance of bluish coloring was observed in the elastogram, consistent with tissues of reduced stiffness (Figure 2).

Quantitatively, liver size showed a significant effect for categorical variables (p = 0.0225), with a 69% probability that the exposed group would manifest hepatomegaly. Additionally, an effect of echotexture (p = 0.0523) was observed, with a 76% probability that the exposed group would exhibit heterogeneous echotexture.

Echogenicity (p = 0.2154), contour (p = 0.1192), and biochemical changes (p = 0.7007) did not differ significantly between the control and exposed groups.

Stiffness (p = 0.0490) differed between the exposed and control groups. The mean shear wave velocity was 2.7140 m/s in the control group and 1.5550 m/s in the exposed group (Figure 2).

In the present study, different liver depth produced no significant difference in the B-mode ultrasonographic assessment between groups (p = 0.1820).

Age influenced hepatic echogenicity (p = 0.0410), with a 65% probability that dogs over 5 years of age would exhibit changes in echogenicity.

The sample size was insufficient for statistical analysis of the categorical variables associated with the level of exposure.

## Discussion

The exposed animals showed a high probability of having a heterogeneous and enlarged liver. Variations in echotexture may be associated with varying levels of liver fibrosis, which may be related to relatively chronic conditions, such as exposure to cigarette smoke compounds (Boisclair et al., 2001; Lawrence & Steiner, 2017). However, inflammatory or infiltrative etiologies cannot be ruled out as causes of hepatic fibrosis.

Diffuse liver enlargement may be observed subjectively or when the borders of the liver become rounded and its margins extend beyond their usual limits, often encroaching upon adjacent structures. This finding may be associated with steroid-induced liver disease, hepatitis and/or cholangitis, lipidosis, neoplasms, passive congestion, or amyloidosis (Larson, 2007; Penninck & d’Anjou, 2015). In this context, the findings of increased liver size in the present study may be correlated with possible hepatitis secondary to exposure to pro-inflammatory cytokines in cigarette smoke (El-Zayadi, 2006). The increase in liver size may also be associated with hepatic congestion. This condition may result from pulmonary hypertension induced by smoke inhalation (Wright & Churg, 1991).

A congestive condition is frequently accompanied by the dilation of hepatic vessels, which enhances their ultrasonographic visualization (Nyland & Mattoon, 2020; Penninck & d’Anjou, 2015). Conversely, in the present study, the detection of portal hepatic vessels was hindered. This can be associated with sinusoidal distention and increased intrahepatic vascular pressure, which can alter the hepatic parenchyma and hinder the ultrasonographic detection of vasculature. Fatty infiltration can also compromise the ultrasonographic evaluation of the intrahepatic vessels by increasing the echogenicity of the hepatic parenchyma (Nyland & Mattoon, 2020; Penninck & d’Anjou, 2015).

In elastographic studies conducted on human breast tissue (Goddi et al., 2012), liver (Ou et al., 2019), and canine kidneys (Moraes et al., 2025), increased tissue stiffness was observed in patients who smoked or were exposed to secondhand smoke. Conversely, in the present study, the group of exposed dogs showed a less rigid hepatic tissue than the control group. We hypothesized that the low stiffness could be associated with possible hepatic congestion resulting from pulmonary hypertension. Inflammation induced by smoke inhalation, which releases vasoactive substances and proteolytic enzymes, may cause pulmonary hypertension (Wright & Churg, 1991). This condition is characterized by increased pressure in the pulmonary circulation and affects the right ventricle, potentially leading to right-sided heart failure. Owing to its proximity to the right ventricle, the liver is the first organ affected by ventricular failure resulting from pulmonary hypertension (Nickel et al., 2021), potentially developing congestive liver disease (Eipel et al., 2010). Assessment of the pulmonary parenchyma was not encompassed within the present study; however, it may be included in future studies. The decreased stiffness of the hepatic parenchyma could also be associated with fatty infiltration. This hypothesis corroborates with the observation that fatty liver disease is negatively affected by cigarette smoke owing to the development of oxidative stress (Azzalini et al., 2010) and metabolic syndrome (where insulin resistance leads to visceral fat deposition) (Ong et al., 2008).

Increased echogenicity of the hepatic parenchyma is frequently associated with fibrosis or fatty infiltration and is caused by increased reflection and scattering of ultrasound waves within the tissue (Nyland & Mattoon, 2020; Penninck & d’Anjou, 2015). Tissue stiffness is associated with the mechanical response of the tissue to deformation. It may be increased in conditions such as fibrosis or neoplasia, where collagen and extracellular matrix deposition occurs (Barr et al., 2015; Holdsworth et al., 2014). In the present study, the increased echogenicity evaluated qualitatively was accompanied by a decreased stiffness of the hepatic parenchyma. This condition is evident in cases of edema or acute inflammation (Dillman et al., 2015; Holdsworth et al., 2014), fatty infiltration (Berzigotti et al., 2021; Ferraioli et al., 2014), and degeneration or necrosis (Barr et al., 2015; Nyland & Mattoon, 2020), where there is an elevated reflection of the sound waves with no collagen or extracellular matrix deposition.

The other ultrasound variables, influence of biochemical alterations, and effect of different depths showed similar statistical results between the two groups. Similarly, the level of exposure to cigarette smoke could not be statistically evaluated owing to the small number of animals analyzed. This limitation hindered the performance of statistical analyses and may restrict the extrapolation of the findings to broader populations.

Regarding age, animals over 5 years of age were relatively likely to exhibit hepatic parenchyma with altered echogenicity. The literature describes a relationship between increasing age and the occurrence of pathological processes, such as the presence of fatty infiltration or cirrhosis (Silva et al., 2007). These hepatic changes are associated with increased parenchymal echogenicity (Partington & Biller, 1995).

Some factors may compromise the data analysis. First, the small number of participants makes it difficult to generalize the results to relatively large groups. Second, the lack of standardization regarding breed is a limiting factor; different breeds may exhibit non-pathological differences in liver tissue stiffness. Healthy brachycephalic dogs may exhibit greater tissue stiffness than mesocephalic dogs (Facin et al., 2020). Third, the standardization of specific liver scanning windows is lacking. Non-pathological stiffness measured using the subcostal window was higher than that measured using the intercostal window (Cha et al., 2022).

Despite their efficacy, ultrasonography and elastography exhibited technical limitations in liver evaluation during the examination of the 23 animals. These limitations included difficulties with intercostal positioning, variation in the size and organ depth of the animals, and interference from structures adjacent to the examined area. The intercostal positioning reduced the acoustic window, thereby potentially compromising the complete B-mode evaluation of the liver and elastography measurements, particularly owing to proximity to the costal margin. We also observed limitations related to the size and frequency of the transducers compared with the acoustic windows available in dogs of different sizes. Finally, the anatomical proximity of the liver to the stomach and duodenum normally generated artifacts resulting from gas or motility. Evaluations affected by such limitations were disregarded in this study.

The small number of patients included in this study, along with the aforementioned limiting factors, rendered the data inadequate for deriving statistically significant results that can be reliably generalized to relatively large populations. Despite these limitations, variations in echogenicity and depth clearly affected the B-mode ultrasonographic evaluation of the liver. These factors contributed to the attenuation of the ultrasound beam in the distal (deeper) regions of the organ, which further complicated the visualization of the hepatic and portal venous structures on B-mode imaging (Tchelepi et al., 2002; Zwiebel, 1995), as well as in elastographic assessment.

## Conclusion

The elastographic findings of reduced liver stiffness in the exposed group were potentially associated with hepatic congestion owing to pulmonary hypertension or fatty infiltration associated with cigarette smoke exposition. However, the present study lacks histopathological testing or comprehensive cardiopulmonary evaluation to confirm these hypotheses. In the B-mode evaluation, exposed dogs showed larger and more and heterogeneous livers than the animals of the control group. These findings confirm the impairment of liver health and reinforce the importance of further investigations using large and homogeneous samples to further elucidate the factors at play in this scenario. Nevertheless, the data obtained demonstrate the impact of passive smoking on the health of pet dogs.
